# The Treatment of Adhesions

**Published:** 1907-04-20

**Authors:** 


### Points in Treatment.

### THE TREATMENT OF ADHESIONS:

### By Thiosinamine and Fibrolysin.

Thiosinamine, we are told, is a derivative of the ethereal oil of mustard (*Sinapis*); it is an allyl-thio-urea compound in which the allyl radicle is attached to an amido group of the urea. Fibrolysin is the proprietary name for what is apparently a combination of thiosinamine and sodium salicylate, soluble in water. The proprietary name is derived from the effect of the compound—namely, the power it seems to have of resolving fibrous tissue in some way.

There is one great objection to the use of either drug—the fact that hypodermic administration is essential. Hypodermic medication is upon the increase, but there can scarcely be any doubt that no medical man would resort to subcutaneous injections if the same results could be obtained by administering the drugs either by the mouth or by the rectum. Notwithstanding this serious objection, there are numbers of cases in which thiosinamine or its sodium salicylate compound are likely to receive extensive trial, for they are cases in which no other form of medical treatment has yet succeeded, and in which even surgery has failed. Every doctor knows the misery produced by perigastric adhesions, unrelieved by gastrojejunostomy; by appendicular adhesions; by perimetritic adhesions; by fibrous stricture of the urethra; by periarticular adhesions and pseudo-ankylosis due to such troubles as gonorrhœal rheumatism; by fibrous stenosis of the œsophagus; and so on. If the drugs under discussion can do no more than relieve these troubles without actually curing them—even if the relief is obtained in but a few cases out of the many that are likely to be treated by them—they will be welcomed. There is already a considerable amount of evidence to show that thiosinamine and its derivative really do lead to some absorption of fibrous tissue. The reports come chiefly from the Continent, where the drugs seem to have been tried and tested since 1892. In England they have attracted notice much more recently, but now they are being tried here too, and it seems that the benefits described by such great authorities as Professor Hebra, Professor Neisser, Professor Ewald, and Dr. Baumstark are being confirmed.

#### What it Does.

The position of affairs seems to be this: No ill results are yet recorded; in more cases than not the result is nil; in many patients, on the other hand, there is marked subjective improvement, with diminution of the pains, increase in weight, and ability to return to the ordinary duties of life; in a few cases the results have been almost marvellous. Amongst the latter, a case of Professor Neisser's may be quoted: A man swallowed some strong caustic soda when he was 22 years old; stenosis of the œsophagus resulted, and the stricture became so complete that no bougie could be passed through it; not even fluids could be got down, and a gastrostomy was performed. The œsophageal stricture persisted for eight years, notwithstanding all the efforts of eminent surgeons and physicians to overcome it. Thiosinamine injections were then resorted to, and given every second day. The stricture began to soften forthwith; after the third injection it was found possible to get a small sound through the site of stenosis; after the twenty-fourth injection the largest size of bougie could readily be passed, and the patient was able to eat anything, and swallow large mouthfuls without any difficulty at all. He had had no return of the stenosis a year later. It may naturally be urged that the cure was fortuitous, and not really assisted by the thiosinamine; but those who saw the result would be quite certain to use the thiosinamine in other cases. Seeing that the stricture had been absolute, and had existed for eight years in spite of every effort to overcome it, the argument is certainly in favour of the relief from thiosinamine being rather *propter hoc* than merely *post hoc*.

#### Indications for its Use.

In any case, the drug is well worthy of trial in other similar instances. The indication for its use is the existence of cicatricial fibrous tissue which we have no other means of getting rid of. It is quite useless, it seems, in cases of neoplasm; at any rate, malignant neoplasm. All observers are unanimous in advising it only for cases in which the cicatricial process is mature and old; in cases of recent inflammation it is liable to increase the trouble rather than diminish it, owing to the lighting up of the dormant inflammation. One would hope that relief could be obtained from it in cases of thickened pleura, adherent pericardium, chronic mediastinitis, and so on, in addition to the other conditions that have been mentioned above.

#### Dosage.

The dose of thiosinamine usually employed is 3 grains (0.2 gram). Fibrolysin is made up in sealed glass bulbs, the contents of each being the dose for one injection. Thiosinamine may be prepared for injection by mixing it with sterilised glycerine and distilled water in the following proportions:—

| | |
|---|---|
| Thiosinanime ... ... ... ... | 2 parts |
| Glycerine ... ... ... ... | 8 parts |
| Distilled water ... ... ... ... | 10 parts |

The injection may be given with an ordinary hypodermic needle and syringe, all the usual aseptic precautions being observed; it may be made into the subcutaneous tissues of the arm, of the abdomen, or any part of the body; that is to say, it does not seem necessary to inject it in the neighbourhood of the cicatricial tissue which it is desired to resolve. The dose is repeated every second day for two or three months.

